# PREFERENCE FOR DIGITAL DEVICES AND PATIENT-GENERATED HEALTH DATA UTILITY IN OLDER PATIENTS AFTER LUNG CANCER SURGERY

**DOI:** 10.1093/geroni/igae098.3556

**Published:** 2024-12-31

**Authors:** Mona Choi, Yeonju Kim, Hyeonmi Cho, Yesol Kim

**Affiliations:** Yonsei University, Seoul, Republic of Korea; Yonsei University, Seoul, Republic of Korea; Yonsei University, Seoul, Republic of Korea; Yonsei University, Seoul, Republic of Korea

## Abstract

This study aimed to investigate the preference for digital devices and the utility of patient-generated health data (PGHD) among older patients who have undergone lung cancer surgery. A cross-sectional descriptive study included patients at least four weeks after lung cancer surgery and excluded those with cognitive impairments. Participants in this study were recruited from a tertiary hospital outpatient clinic and conducted using a structured questionnaire: 1) patient’s general characteristics; 2) experience and duration of use of digital devices; 3) preference for wearable or attachable digital devices for PGHD collection; and 4) PGHD usability. Descriptive and bivariate analyses were performed. 135 patients were analyzed (age = 70.19 ± 11.80 years; 47.0% female). Smartphones (94.1%) were the most experienced digital devices, and the period of use varied from 4 months to 15 years. The wristwatch type was the most preferred wearable digital device (61.5%), and men preferred the belt type. Depending on the level of education, there was a difference in preference between the watch type and the skin patch type, and there was a difference in preference for the glasses type depending on the quality of sleep. The most informative PGHDs were lung function (91.1%), blood pressure (83.7%), and step count (83.7%). The usefulness of PGHD, such as drug side effects and social relationships, differed depending on the number of drugs taken. This study may help healthcare providers select appropriate digital health devices and utilize useful PGHD to improve health outcomes among older patients after lung cancer surgery.

